# Influence of polygenic risk for schizophrenia and cannabis use on cognition

**DOI:** 10.1192/j.eurpsy.2025.1199

**Published:** 2025-08-26

**Authors:** A. Flores, G. Cano, I. Zorrilla, S. Amoretti, E. Vieta, L. Pina-Camacho, A. M. Sánchez-Torres, D. Bergé, S. Mas, A. González-Pinto

**Affiliations:** 1 Hospital Santiago Apóstol; 2 BIOARABA; 3 CIBERSAM, Vitoria-Gasteiz; 4 Hospital Clínic, Barcelona; 5 Hospital Gregorio Marañón, Madrid; 6 Complejo Hospitalario Navarra, Pamplona; 7 Hospital del Mar; 8 CIBERSAM, Barcelona, Spain

## Abstract

**Introduction:**

There is increasing scientific evidence linking the influence of cannabis on the onset of a first psychotic episode (FEP). However, this association may not always be one-way when studying the relationship between cannabis use and cognition.

The role of polygenic risk score for Schizophrenia (PRS-SZ) and lifetime cannabis initiation (PRSCI) and cannabis use disorder (PRSCUD) use remains unresolved. For this reason, a study was carried out to clarify how these factors are related to each other.

**Objectives:**

To study the interaction between genetic risk and environmental factors

To analyze the relationship between polygenic risk scores and clinical features

To determine the impact of cannabis use on cognitive performance

**Methods:**

A neuropsychological study was carried out on a sample of 138 cannabis users, divided into cases and controls according to the existence of a FEP. This assessment included domains of processing speed, verbal memory, attention, working memory, executive function, social cognition, and determination of premorbid IQ, using validated tests.

Three GWAS summary statistics were used to calculate individual PRS conferring risk for SZ, CI and CUD. PRS were constructed using PRS-CS.

The results obtained were then statistically correlated with the polygenic risk score for schizophrenia, cannabis use disorder, and lifetime cannabis use, respectively.

**Results:**

The patient group presented worse processing speed as the risk for schizophrenia increased. In the case of PRS-CU, an opposite effect was evident. For verbal memory, a higher PRS-CUD was associated with poorer performance. Cannabis consumers with lifetime risk presented better executive function than consumers with lower genetic risk.

**Conclusions:**

The relationship between PRS for Schizophrenia and cannabis use remains uncertain. However, different clinical profiles can be determined thanks to the cognitive assessment.

**Disclosure of Interest:**

None Declared

